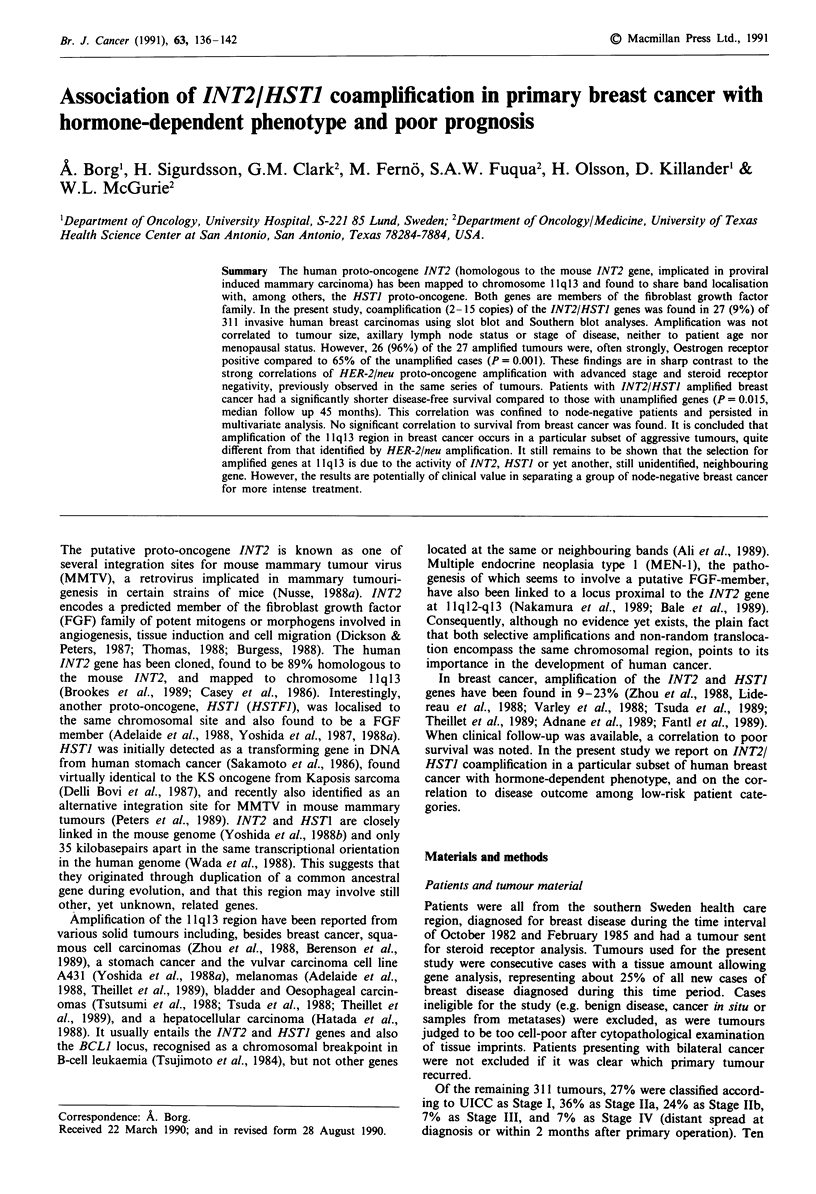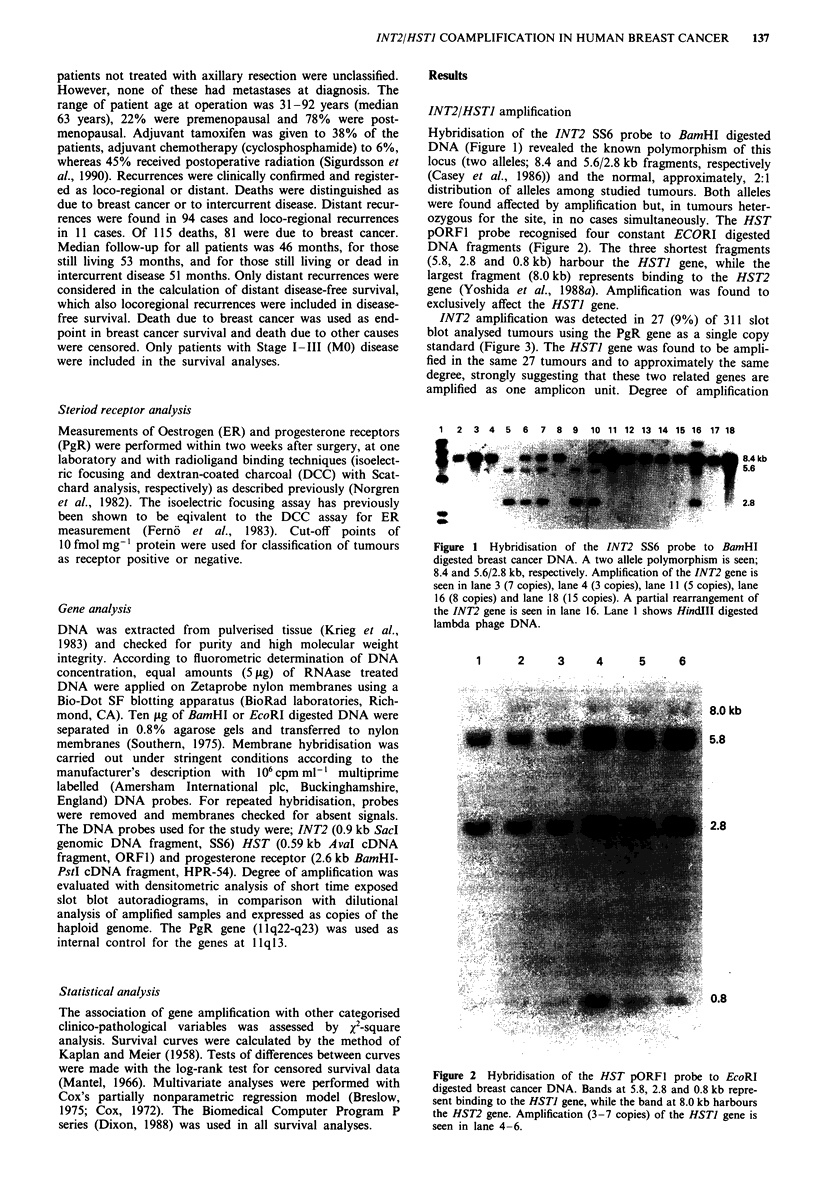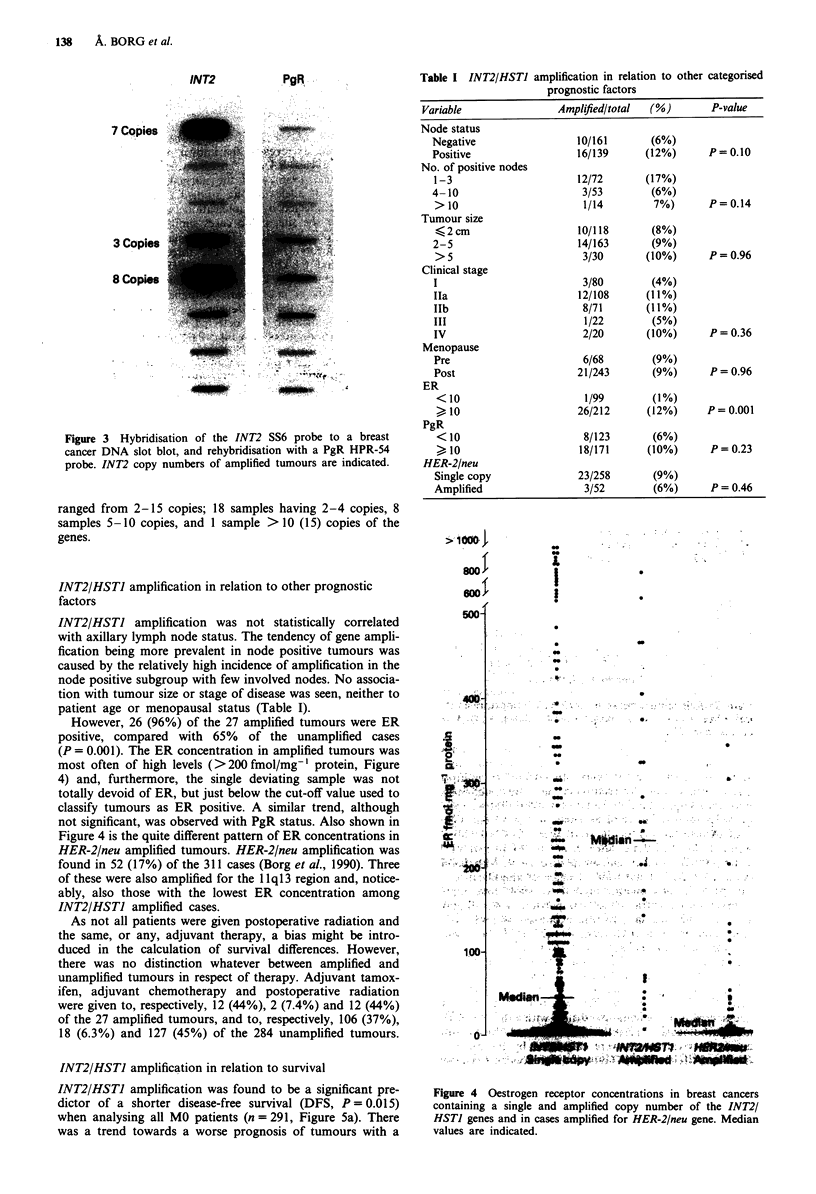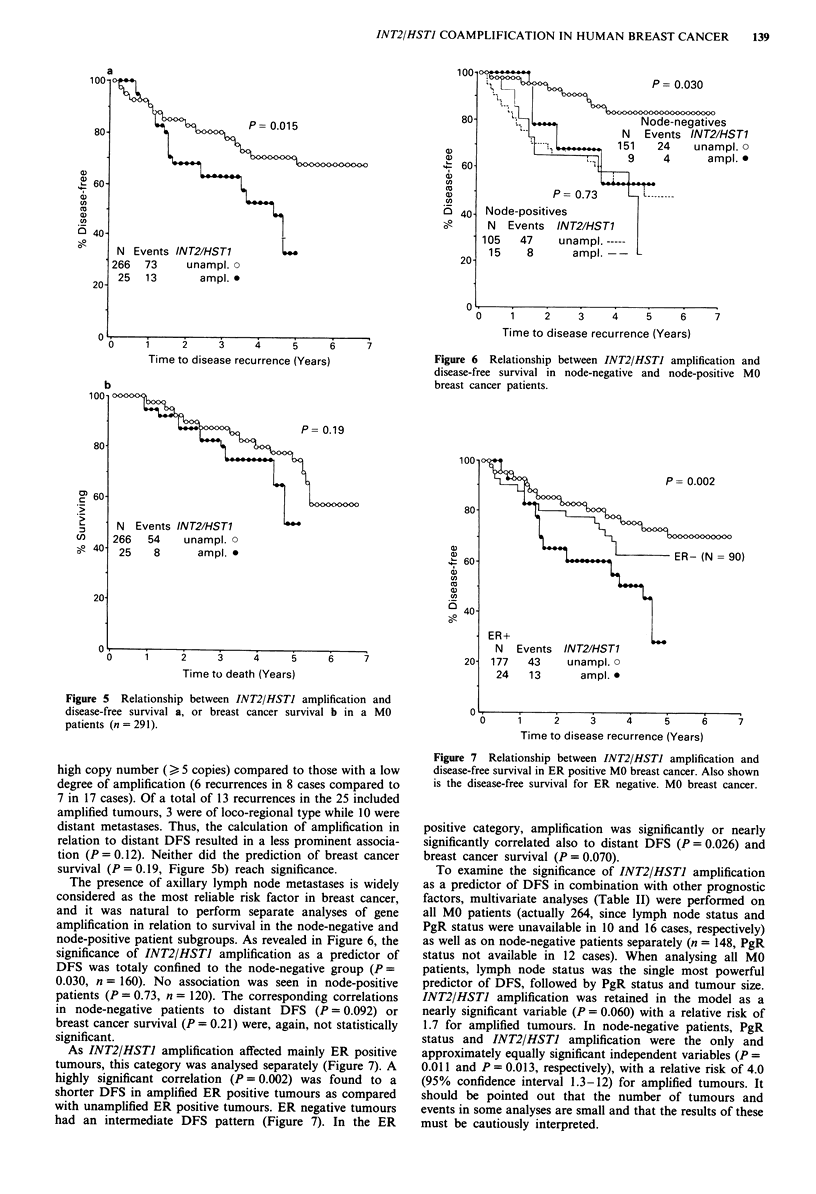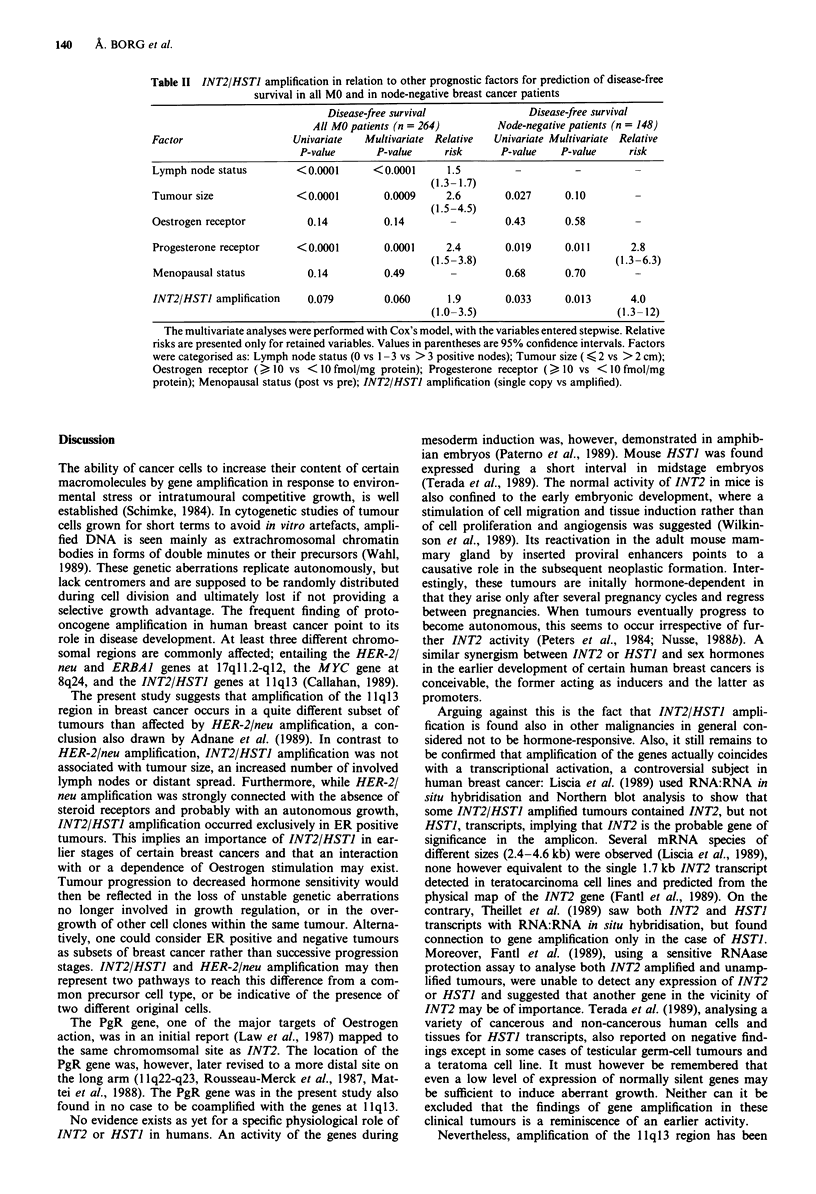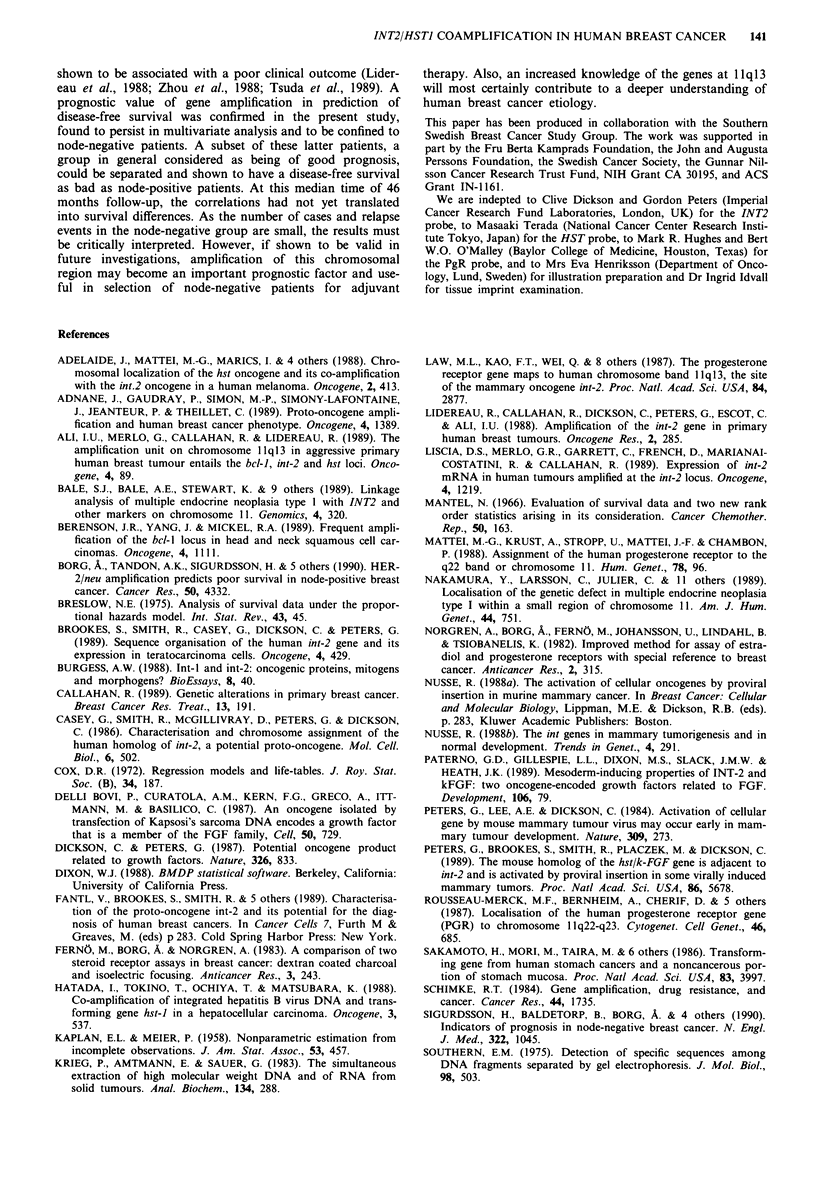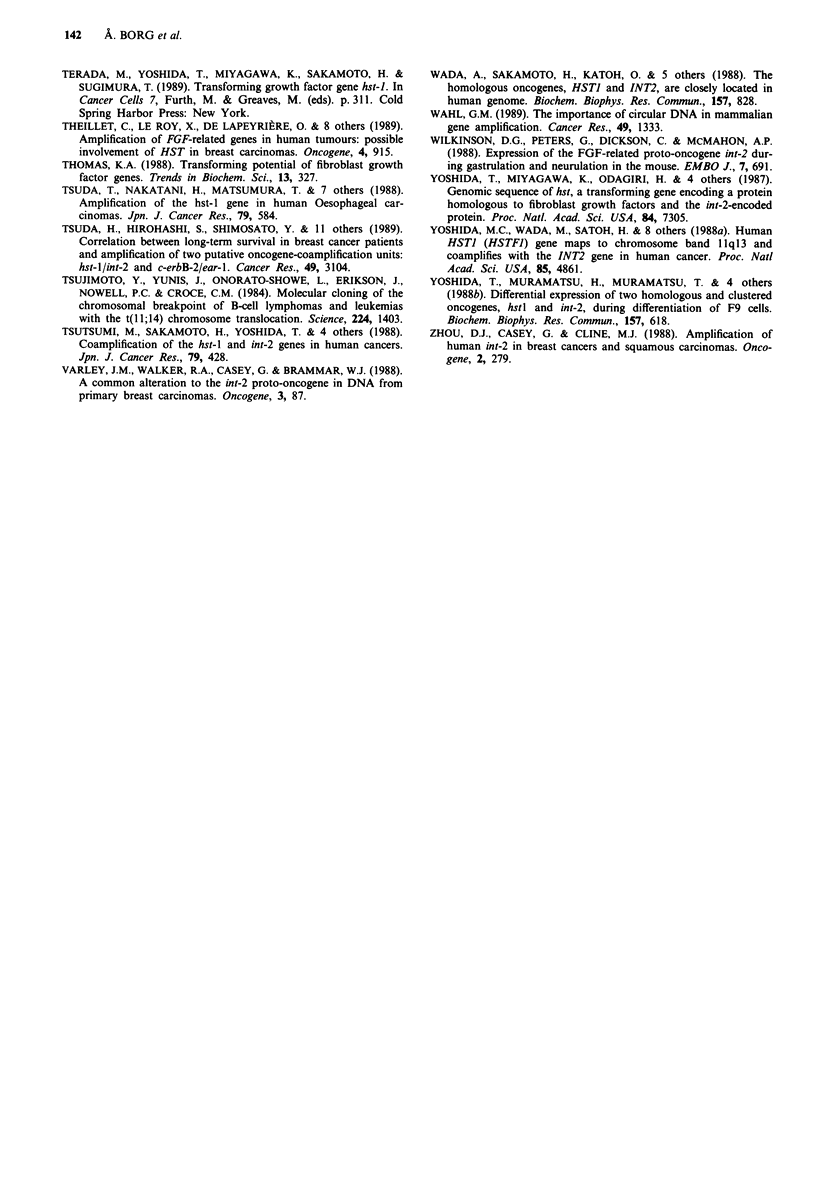# Association of INT2/HST1 coamplification in primary breast cancer with hormone-dependent phenotype and poor prognosis.

**DOI:** 10.1038/bjc.1991.28

**Published:** 1991-01

**Authors:** A. Borg, H. Sigurdsson, G. M. Clark, M. Fernö, S. A. Fuqua, H. Olsson, D. Killander, W. L. McGurie

**Affiliations:** Department of Oncology, University Hospital, Lund, Sweden.

## Abstract

**Images:**


Br. J. Cancer (1991), 63, 136-142 © Macmillan Press Ltd., 1991

### Association of *INT2/HST1* coamplification in primary breast cancer with hormone-dependent phenotype and poor prognosis

Å. Borg[1], H. Sigurdsson, G.M. Clark[2], M. Fernö, S.A.W. Fuqua[2], H. Olsson, D. Killander[1] & W.L. McGurie[2]

[1]*Department of Oncology, University Hospital, S-221 85 Lund, Sweden;* [2]*Department of Oncology/Medicine, University of Texas Health Science Center at San Antonio, San Antonio, Texas 78284-7884, USA.*

**Summary** The human proto-oncogene *INT2* (homologous to the mouse *INT2* gene, implicated in proviral induced mammary carcinoma) has been mapped to chromosome 11q13 and found to share band localisation with, among others, the *HST1* proto-oncogene. Both genes are members of the fibroblast growth factor family. In the present study, coamplification (2–15 copies) of the *INT2/HST1* genes was found in 27 (9%) of 311 invasive human breast carcinomas using slot blot and Southern blot analyses. Amplification was not correlated to tumour size, axillary lymph node status or stage of disease, neither to patient age nor menopausal status. However, 26 (96%) of the 27 amplified tumours were, often strongly, Oestrogen receptor positive compared to 65% of the unamplified cases ($P = 0.001$). These findings are in sharp contrast to the strong correlations of *HER-2/neu* proto-oncogene amplification with advanced stage and steroid receptor negativity, previously observed in the same series of tumours. Patients with *INT2/HST1* amplified breast cancer had a significantly shorter disease-free survival compared to those with unamplified genes ($P = 0.015$, median follow up 45 months). This correlation was confined to node-negative patients and persisted in multivariate analysis. No significant correlation to survival from breast cancer was found. It is concluded that amplification of the 11q13 region in breast cancer occurs in a particular subset of aggressive tumours, quite different from that identified by *HER-2/neu* amplification. It still remains to be shown that the selection for amplified genes at 11q13 is due to the activity of *INT2*, *HST1* or yet another, still unidentified, neighbouring gene. However, the results are potentially of clinical value in separating a group of node-negative breast cancer for more intense treatment.

The putative proto-oncogene *INT2* is known as one of several integration sites for mouse mammary tumour virus (MMTV), a retrovirus implicated in mammary tumourigenesis in certain strains of mice (Nusse, 1988*a*). *INT2* encodes a predicted member of the fibroblast growth factor (FGF) family of potent mitogens or morphogens involved in angiogenesis, tissue induction and cell migration (Dickson & Peters, 1987; Thomas, 1988; Burgess, 1988). The human *INT2* gene has been cloned, found to be 89% homologous to the mouse *INT2*, and mapped to chromosome 11q13 (Brookes *et al.*, 1989; Casey *et al.*, 1986). Interestingly, another proto-oncogene, *HST1* (*HSTF1*), was localised to the same chromosomal site and also found to be a FGF member (Adelaide *et al.*, 1988, Yoshida *et al.*, 1987, 1988*a*). *HST1* was initially detected as a transforming gene in DNA from human stomach cancer (Sakamoto *et al.*, 1986), found virtually identical to the KS oncogene from Kaposis sarcoma (Delli Bovi *et al.*, 1987), and recently also identified as an alternative integration site for MMTV in mouse mammary tumours (Peters *et al.*, 1989). *INT2* and *HST*1 are closely linked in the mouse genome (Yoshida *et al.*, 1988*b*) and only 35 kilobasepairs apart in the same transcriptional orientation in the human genome (Wada *et al.*, 1988). This suggests that they originated through duplication of a common ancestral gene during evolution, and that this region may involve still other, yet unknown, related genes.

Amplification of the 11q13 region have been reported from various solid tumours including, besides breast cancer, squamous cell carcinomas (Zhou *et al.*, 1988, Berenson *et al.*, 1989), a stomach cancer and the vulvar carcinoma cell line A431 (Yoshida *et al.*, 1988*a*), melanomas (Adelaide *et al.*, 1988, Theillet *et al.*, 1989), bladder and Oesophageal carcinomas (Tsutsumi *et al.*, 1988; Tsuda *et al.*, 1988; Theillet *et al.*, 1989), and a hepatocellular carcinoma (Hatada *et al.*, 1988). It usually entails the *INT2* and *HST1* genes and also the *BCL1* locus, recognised as a chromosomal breakpoint in B-cell leukaemia (Tsujimoto *et al.*, 1984), but not other genes located at the same or neighbouring bands (Ali *et al.*, 1989). Multiple endocrine neoplasia type 1 (MEN-1), the pathogenesis of which seems to involve a putative FGF-member, have also been linked to a locus proximal to the *INT2* gene at 11q12-q13 (Nakamura *et al.*, 1989; Bale *et al.*, 1989). Consequently, although no evidence yet exists, the plain fact that both selective amplifications and non-random translocation encompass the same chromosomal region, points to its importance in the development of human cancer.

In breast cancer, amplification of the *INT2* and *HST1* genes have been found in 9–23% (Zhou *et al.*, 1988, Lidereau *et al.*, 1988; Varley *et al.*, 1988; Tsuda *et al.*, 1989; Theillet *et al.*, 1989; Adnane *et al.*, 1989; Fantl *et al.*, 1989). When clinical follow-up was available, a correlation to poor survival was noted. In the present study we report on *INT2/HST1* coamplification in a particular subset of human breast cancer with hormone-dependent phenotype, and on the correlation to disease outcome among low-risk patient categories.

### Materials and methods

### *Patients and tumour material*

Patients were all from the southern Sweden health care region, diagnosed for breast disease during the time interval of October 1982 and February 1985 and had a tumour sent for steroid receptor analysis. Tumours used for the present study were consecutive cases with a tissue amount allowing gene analysis, representing about 25% of all new cases of breast disease diagnosed during this time period. Cases ineligible for the study (e.g. benign disease, cancer *in situ* or samples from metatases) were excluded, as were tumours judged to be too cell-poor after cytopathological examination of tissue imprints. Patients presenting with bilateral cancer were not excluded if it was clear which primary tumour recurred.

Of the remaining 311 tumours, 27% were classified according to UICC as Stage I, 36% as Stage IIa, 24% as Stage IIb, 7% as Stage III, and 7% as Stage IV (distant spread at diagnosis or within 2 months after primary operation). Ten

Correspondence: Å. Borg.
Received 22 March 1990; and in revised form 28 August 1990.

patients not treated with axillary resection were unclassified. However, none of these had metastases at diagnosis. The range of patient age at operation was 31–92 years (median 63 years), 22% were premenopausal and 78% were postmenopausal. Adjuvant tamoxifen was given to 38% of the patients, adjuvant chemotherapy (cyclosphosphamide) to 6%, whereas 45% received postoperative radiation (Sigurdsson *et al.*, 1990). Recurrences were clinically confirmed and registered as loco-regional or distant. Deaths were distinguished as due to breast cancer or to intercurrent disease. Distant recurrences were found in 94 cases and loco-regional recurrences in 11 cases. Of 115 deaths, 81 were due to breast cancer. Median follow-up for all patients was 46 months, for those still living 53 months, and for those still living or dead in intercurrent disease 51 months. Only distant recurrences were considered in the calculation of distant disease-free survival, which also locoregional recurrences were included in disease-free survival. Death due to breast cancer was used as end-point in breast cancer survival and death due to other causes were censored. Only patients with Stage I–III (M0) disease were included in the survival analyses.

### *Steriod receptor analysis*

Measurements of Oestrogen (ER) and progesterone receptors (PgR) were performed within two weeks after surgery, at one laboratory and with radioligand binding techniques (isoelectric focusing and dextran-coated charcoal (DCC) with Scatchard analysis, respectively) as described previously (Norgren *et al.*, 1982). The isoelectric focusing assay has previously been shown to be eqivalent to the DCC assay for ER measurement (Fernö *et al.*, 1983). Cut-off points of 10 fmol mg$^{-1}$ protein were used for classification of tumours as receptor positive or negative.

### *Gene analysis*

DNA was extracted from pulverised tissue (Krieg *et al.*, 1983) and checked for purity and high molecular weight integrity. According to fluorometric determination of DNA concentration, equal amounts (5 μg) of RNAase treated DNA were applied on Zetaprobe nylon membranes using a Bio-Dot SF blotting apparatus (BioRad laboratories, Richmond, CA). Ten μg of *Bam*HI or *Eco*RI digested DNA were separated in 0.8% agarose gels and transferred to nylon membranes (Southern, 1975). Membrane hybridisation was carried out under stringent conditions according to the manufacturer's description with $10^6$ cpm ml$^{-1}$ multiprime labelled (Amersham International plc, Buckinghamshire, England) DNA probes. For repeated hybridisation, probes were removed and membranes checked for absent signals. The DNA probes used for the study were; *INT2* (0.9 kb *Sac*I genomic DNA fragment, SS6) *HST* (0.59 kb *Ava*I cDNA fragment, ORF1) and progesterone receptor (2.6 kb *Bam*HI-*Pst*I cDNA fragment, HPR-54). Degree of amplification was evaluated with densitometric analysis of short time exposed slot blot autoradiograms, in comparison with dilutional analysis of amplified samples and expressed as copies of the haploid genome. The PgR gene (11q22-q23) was used as internal control for the genes at 11q13.

### *Statistical analysis*

The association of gene amplification with other categorised clinico-pathological variables was assessed by $\chi^2$-square analysis. Survival curves were calculated by the method of Kaplan and Meier (1958). Tests of differences between curves were made with the log-rank test for censored survival data (Mantel, 1966). Multivariate analyses were performed with Cox's partially nonparametric regression model (Breslow, 1975; Cox, 1972). The Biomedical Computer Program P series (Dixon, 1988) was used in all survival analyses.

### Results

### *INT2/HST1* amplification

Hybridisation of the *INT2* SS6 probe to *Bam*HI digested DNA (Figure 1) revealed the known polymorphism of this locus (two alleles; 8.4 and 5.6/2.8 kb fragments, respectively (Casey *et al.*, 1986)) and the normal, approximately, 2:1 distribution of alleles among studied tumours. Both alleles were found affected by amplification but, in tumours heterozygous for the site, in no cases simultaneously. The *HST* pORF1 probe recognised four constant *ECO*RI digested DNA fragments (Figure 2). The three shortest fragments (5.8, 2.8 and 0.8 kb) harbour the *HST1* gene, while the largest fragment (8.0 kb) represents binding to the *HST2* gene (Yoshida *et al.*, 1988*a*). Amplification was found to exclusively affect the *HST1* gene.

*INT2* amplification was detected in 27 (9%) of 311 slot blot analysed tumours using the PgR gene as a single copy standard (Figure 3). The *HST1* gene was found to be amplified in the same 27 tumours and to approximately the same degree, strongly suggesting that these two related genes are amplified as one amplicon unit. Degree of amplification

**Figure 1** Hybridisation of the *INT2* SS6 probe to *Bam*HI digested breast cancer DNA. A two allele polymorphism is seen; 8.4 and 5.6/2.8 kb, respectively. Amplification of the *INT2* gene is seen in lane 3 (7 copies), lane 4 (3 copies), lane 11 (5 copies), lane 16 (8 copies) and lane 18 (15 copies). A partial rearrangement of the *INT2* gene is seen in lane 16. Lane 1 shows *Hin*dIII digested lambda phage DNA.

**Figure 2** Hybridisation of the *HST* pORF1 probe to *Eco*RI digested breast cancer DNA. Bands at 5.8, 2.8 and 0.8 kb represent binding to the *HST1* gene, while the band at 8.0 kb harbours the *HST2* gene. Amplification (3–7 copies) of the *HST1* gene is seen in lane 4–6.

**Figure 3** Hybridisation of the *INT2* SS6 probe to a breast cancer DNA slot blot, and rehybridisation with a PgR HPR-54 probe. *INT2* copy numbers of amplified tumours are indicated.

ranged from 2–15 copies; 18 samples having 2–4 copies, 8 samples 5–10 copies, and 1 sample >10 (15) copies of the genes.

*INT2/HST1* amplification in relation to other prognostic factors

*INT2/HST1* amplification was not statistically correlated with axillary lymph node status. The tendency of gene amplification being more prevalent in node positive tumours was caused by the relatively high incidence of amplification in the node positive subgroup with few involved nodes. No association with tumour size or stage of disease was seen, neither to patient age or menopausal status (Table I).

However, 26 (96%) of the 27 amplified tumours were ER positive, compared with 65% of the unamplified cases ($P = 0.001$). The ER concentration in amplified tumours was most often of high levels (>200 fmol/mg$^{-1}$ protein, Figure 4) and, furthermore, the single deviating sample was not totally devoid of ER, but just below the cut-off value used to classify tumours as ER positive. A similar trend, although not significant, was observed with PgR status. Also shown in Figure 4 is the quite different pattern of ER concentrations in *HER-2/neu* amplified tumours. *HER-2/neu* amplification was found in 52 (17%) of the 311 cases (Borg *et al.*, 1990). Three of these were also amplified for the 11q13 region and, noticeably, also those with the lowest ER concentration among *INT2/HST1* amplified cases.

As not all patients were given postoperative radiation and the same, or any, adjuvant therapy, a bias might be introduced in the calculation of survival differences. However, there was no distinction whatever between amplified and unamplified tumours in respect of therapy. Adjuvant tamoxifen, adjuvant chemotherapy and postoperative radiation were given to, respectively, 12 (44%), 2 (7.4%) and 12 (44%) of the 27 amplified tumours, and to, respectively, 106 (37%), 18 (6.3%) and 127 (45%) of the 284 unamplified tumours.

*INT2/HST1* amplification in relation to survival

*INT2/HST1* amplification was found to be a significant predictor of a shorter disease-free survival (DFS, $P = 0.015$) when analysing all M0 patients ($n = 291$, Figure 5a). There was a trend towards a worse prognosis of tumours with a

**Table I** *INT2/HST1* amplification in relation to other categorised prognostic factors

| *Variable* | *Amplified/total* | *(%)* | *P-value* |
|---|---|---|---|
| Node status | | | |
| Negative | 10/161 | (6%) | |
| Positive | 16/139 | (12%) | $P = 0.10$ |
| No. of positive nodes | | | |
| 1–3 | 12/72 | (17%) | |
| 4–10 | 3/53 | (6%) | |
| >10 | 1/14 | 7%) | $P = 0.14$ |
| Tumour size | | | |
| ⩽2 cm | 10/118 | (8%) | |
| 2–5 | 14/163 | (9%) | |
| >5 | 3/30 | (10%) | $P = 0.96$ |
| Clinical stage | | | |
| I | 3/80 | (4%) | |
| IIa | 12/108 | (11%) | |
| IIb | 8/71 | (11%) | |
| III | 1/22 | (5%) | |
| IV | 2/20 | (10%) | $P = 0.36$ |
| Menopause | | | |
| Pre | 6/68 | (9%) | |
| Post | 21/243 | (9%) | $P = 0.96$ |
| ER | | | |
| <10 | 1/99 | (1%) | |
| ⩾10 | 26/212 | (12%) | $P = 0.001$ |
| PgR | | | |
| <10 | 8/123 | (6%) | |
| ⩾10 | 18/171 | (10%) | $P = 0.23$ |
| *HER-2/neu* | | | |
| Single copy | 23/258 | (9%) | |
| Amplified | 3/52 | (6%) | $P = 0.46$ |

**Figure 4** Oestrogen receptor concentrations in breast cancers containing a single and amplified copy number of the *INT2/HST1* genes and in cases amplified for *HER-2/neu* gene. Median values are indicated.

**Figure 5** Relationship between *INT2/HST1* amplification and disease-free survival **a**, or breast cancer survival **b** in a M0 patients ($n = 291$).

**Figure 6** Relationship between *INT2/HST1* amplification and disease-free survival in node-negative and node-positive M0 breast cancer patients.

**Figure 7** Relationship between *INT2/HST1* amplification and disease-free survival in ER positive M0 breast cancer. Also shown is the disease-free survival for ER negative. M0 breast cancer.

high copy number ($\geqslant 5$ copies) compared to those with a low degree of amplification (6 recurrences in 8 cases compared to 7 in 17 cases). Of a total of 13 recurrences in the 25 included amplified tumours, 3 were of loco-regional type while 10 were distant metastases. Thus, the calculation of amplification in relation to distant DFS resulted in a less prominent association ($P = 0.12$). Neither did the prediction of breast cancer survival ($P = 0.19$, Figure 5b) reach significance.

The presence of axillary lymph node metastases is widely considered as the most reliable risk factor in breast cancer, and it was natural to perform separate analyses of gene amplification in relation to survival in the node-negative and node-positive patient subgroups. As revealed in Figure 6, the significance of *INT2/HST1* amplification as a predictor of DFS was totaly confined to the node-negative group ($P = 0.030$, $n = 160$). No association was seen in node-positive patients ($P = 0.73$, $n = 120$). The corresponding correlations in node-negative patients to distant DFS ($P = 0.092$) or breast cancer survival ($P = 0.21$) were, again, not statistically significant.

As *INT2/HST1* amplification affected mainly ER positive tumours, this category was analysed separately (Figure 7). A highly significant correlation ($P = 0.002$) was found to a shorter DFS in amplified ER positive tumours as compared with unamplified ER positive tumours. ER negative tumours had an intermediate DFS pattern (Figure 7). In the ER positive category, amplification was significantly or nearly significantly correlated also to distant DFS ($P = 0.026$) and breast cancer survival ($P = 0.070$).

To examine the significance of *INT2/HST1* amplification as a predictor of DFS in combination with other prognostic factors, multivariate analyses (Table II) were performed on all M0 patients (actually 264, since lymph node status and PgR status were unavailable in 10 and 16 cases, respectively) as well as on node-negative patients separately ($n = 148$, PgR status not available in 12 cases). When analysing all M0 patients, lymph node status was the single most powerful predictor of DFS, followed by PgR status and tumour size. *INT2/HST1* amplification was retained in the model as a nearly significant variable ($P = 0.060$) with a relative risk of 1.7 for amplified tumours. In node-negative patients, PgR status and *INT2/HST1* amplification were the only and approximately equally significant independent variables ($P = 0.011$ and $P = 0.013$, respectively), with a relative risk of 4.0 (95% confidence interval 1.3–12) for amplified tumours. It should be pointed out that the number of tumours and events in some analyses are small and that the results of these must be cautiously interpreted.

**Table II** *INT2/HST1* amplification in relation to other prognostic factors for prediction of disease-free survival in all M0 and in node-negative breast cancer patients

| Factor | *Disease-free survival All M0 patients (n = 264)* Univariate P-value | Multivariate P-value | Relative risk | *Disease-free survival Node-negative patients (n = 148)* Univariate P-value | Multivariate P-value | Relative risk |
|---|---|---|---|---|---|---|
| Lymph node status | <0.0001 | <0.0001 | 1.5 (1.3–1.7) | – | – | – |
| Tumour size | <0.0001 | 0.0009 | 2.6 (1.5–4.5) | 0.027 | 0.10 | – |
| Oestrogen receptor | 0.14 | 0.14 | – | 0.43 | 0.58 | – |
| Progesterone receptor | <0.0001 | 0.0001 | 2.4 (1.5–3.8) | 0.019 | 0.011 | 2.8 (1.3–6.3) |
| Menopausal status | 0.14 | 0.49 | – | 0.68 | 0.70 | – |
| *INT2/HST1* amplification | 0.079 | 0.060 | 1.9 (1.0–3.5) | 0.033 | 0.013 | 4.0 (1.3–12) |

The multivariate analyses were performed with Cox's model, with the variables entered stepwise. Relative risks are presented only for retained variables. Values in parentheses are 95% confidence intervals. Factors were categorised as: Lymph node status (0 vs 1–3 vs >3 positive nodes); Tumour size ($\leqslant$2 vs >2 cm); Oestrogen receptor ($\geqslant$10 vs <10 fmol/mg protein); Progesterone receptor ($\geqslant$10 vs <10 fmol/mg protein); Menopausal status (post vs pre); *INT2/HST1* amplification (single copy vs amplified).

### Discussion

The ability of cancer cells to increase their content of certain macromolecules by gene amplification in response to environmental stress or intratumoural competitive growth, is well established (Schimke, 1984). In cytogenetic studies of tumour cells grown for short terms to avoid *in vitro* artefacts, amplified DNA is seen mainly as extrachromosomal chromatin bodies in forms of double minutes or their precursors (Wahl, 1989). These genetic aberrations replicate autonomously, but lack centromers and are supposed to be randomly distributed during cell division and ultimately lost if not providing a selective growth advantage. The frequent finding of proto-oncogene amplification in human breast cancer point to its role in disease development. At least three different chromosomal regions are commonly affected; entailing the *HER-2/neu* and *ERBA1* genes at 17q11.2-q12, the *MYC* gene at 8q24, and the *INT2/HST1* genes at 11q13 (Callahan, 1989).

The present study suggests that amplification of the 11q13 region in breast cancer occurs in a quite different subset of tumours than affected by *HER-2/neu* amplification, a conclusion also drawn by Adnane *et al.* (1989). In contrast to *HER-2/neu* amplification, *INT2/HST1* amplification was not associated with tumour size, an increased number of involved lymph nodes or distant spread. Furthermore, while *HER-2/neu* amplification was strongly connected with the absence of steroid receptors and probably with an autonomous growth, *INT2/HST1* amplification occurred exclusively in ER positive tumours. This implies an importance of *INT2/HST1* in earlier stages of certain breast cancers and that an interaction with or a dependence of Oestrogen stimulation may exist. Tumour progression to decreased hormone sensitivity would then be reflected in the loss of unstable genetic aberrations no longer involved in growth regulation, or in the overgrowth of other cell clones within the same tumour. Alternatively, one could consider ER positive and negative tumours as subsets of breast cancer rather than successive progression stages. *INT2/HST1* and *HER-2/neu* amplification may then represent two pathways to reach this difference from a common precursor cell type, or be indicative of the presence of two different original cells.

The PgR gene, one of the major targets of Oestrogen action, was in an initial report (Law *et al.*, 1987) mapped to the same chromomsomal site as *INT2*. The location of the PgR gene was, however, later revised to a more distal site on the long arm (11q22-q23, Rousseau-Merck *et al.*, 1987, Mattei *et al.*, 1988). The PgR gene was in the present study also found in no case to be coamplified with the genes at 11q13.

No evidence exists as yet for a specific physiological role of *INT2* or *HST1* in humans. An activity of the genes during mesoderm induction was, however, demonstrated in amphibian embryos (Paterno *et al.*, 1989). Mouse *HST1* was found expressed during a short interval in midstage embryos (Terada *et al.*, 1989). The normal activity of *INT2* in mice is also confined to the early embryonic development, where a stimulation of cell migration and tissue induction rather than of cell proliferation and angiogensis was suggested (Wilkinson *et al.*, 1989). Its reactivation in the adult mouse mammary gland by inserted proviral enhancers points to a causative role in the subsequent neoplastic formation. Interestingly, these tumours are initally hormone-dependent in that they arise only after several pregnancy cycles and regress between pregnancies. When tumours eventually progress to become autonomous, this seems to occur irrespective of further *INT2* activity (Peters *et al.*, 1984; Nusse, 1988*b*). A similar synergism between *INT2* or *HST1* and sex hormones in the earlier development of certain human breast cancers is conceivable, the former acting as inducers and the latter as promoters.

Arguing against this is the fact that *INT2/HST1* amplification is found also in other malignancies in general considered not to be hormone-responsive. Also, it still remains to be confirmed that amplification of the genes actually coincides with a transcriptional activation, a controversial subject in human breast cancer: Liscia *et al.* (1989) used RNA:RNA *in situ* hybridisation and Northern blot analysis to show that some *INT2/HST1* amplified tumours contained *INT2*, but not *HST1*, transcripts, implying that *INT2* is the probable gene of significance in the amplicon. Several mRNA species of different sizes (2.4–4.6 kb) were observed (Liscia *et al.*, 1989), none however equivalent to the single 1.7 kb *INT2* transcript detected in teratocarcinoma cell lines and predicted from the physical map of the *INT2* gene (Fantl *et al.*, 1989). On the contrary, Theillet *et al.* (1989) saw both *INT2* and *HST1* transcripts with RNA:RNA *in situ* hybridisation, but found connection to gene amplification only in the case of *HST1*. Moreover, Fantl *et al.* (1989), using a sensitive RNAase protection assay to analyse both *INT2* amplified and unamplified tumours, were unable to detect any expression of *INT2* or *HST1* and suggested that another gene in the vicinity of *INT2* may be of importance. Terada *et al.* (1989), analysing a variety of cancerous and non-cancerous human cells and tissues for *HST1* transcripts, also reported on negative findings except in some cases of testicular germ-cell tumours and a teratoma cell line. It must however be remembered that even a low level of expression of normally silent genes may be sufficient to induce aberrant growth. Neither can it be excluded that the findings of gene amplification in these clinical tumours is a reminiscence of an earlier activity.

Nevertheless, amplification of the 11q13 region has been shown to be associated with a poor clinical outcome (Lidereau *et al.*, 1988; Zhou *et al.*, 1988; Tsuda *et al.*, 1989). A prognostic value of gene amplification in prediction of disease-free survival was confirmed in the present study, found to persist in multivariate analysis and to be confined to node-negative patients. A subset of these latter patients, a group in general considered as being of good prognosis, could be separated and shown to have a disease-free survival as bad as node-positive patients. At this median time of 46 months follow-up, the correlations had not yet translated into survival differences. As the number of cases and relapse events in the node-negative group are small, the results must be critically interpreted. However, if shown to be valid in future investigations, amplification of this chromosomal region may become an important prognostic factor and useful in selection of node-negative patients for adjuvant therapy. Also, an increased knowledge of the genes at 11q13 will most certainly contribute to a deeper understanding of human breast cancer etiology.

This paper has been produced in collaboration with the Southern Swedish Breast Cancer Study Group. The work was supported in part by the Fru Berta Kamprads Foundation, the John and Augusta Perssons Foundation, the Swedish Cancer Society, the Gunnar Nilsson Cancer Research Trust Fund, NIH Grant CA 30195, and ACS Grant IN-1161.

We are indepted to Clive Dickson and Gordon Peters (Imperial Cancer Research Fund Laboratories, London, UK) for the *INT2* probe, to Masaaki Terada (National Cancer Center Research Institute Tokyo, Japan) for the *HST* probe, to Mark R. Hughes and Bert W.O. O'Malley (Baylor College of Medicine, Houston, Texas) for the PgR probe, and to Mrs Eva Henriksson (Department of Oncology, Lund, Sweden) for illustration preparation and Dr Ingrid Idvall for tissue imprint examination.

### References

ADELAIDE, J., MATTEI, M.-G., MARICS, I. & 4 others (1988). Chromosomal localization of the *hst* oncogene and its co-amplification with the *int.2* oncogene in a human melanoma. *Oncogene*, **2**, 413.

ADNANE, J., GAUDRAY, P., SIMON, M.-P., SIMONY-LAFONTAINE, J., JEANTEUR, P. & THEILLET, C. (1989). Proto-oncogene amplification and human breast cancer phenotype. *Oncogene*, **4**, 1389.

ALI, I.U., MERLO, G., CALLAHAN, R. & LIDEREAU, R. (1989). The amplification unit on chromosome 11q13 in aggressive primary human breast tumour entails the *bcl-1*, *int-2* and *hst* loci. *Oncogene*, **4**, 89.

BALE, S.J., BALE, A.E., STEWART, K. & 9 others (1989). Linkage analysis of multiple endocrine neoplasia type 1 with *INT2* and other markers on chromosome 11. *Genomics*, **4**, 320.

BERENSON, J.R., YANG, J. & MICKEL, R.A. (1989). Frequent amplification of the *bcl*-1 locus in head and neck squamous cell carcinomas. *Oncogene*, **4**, 1111.

BORG, Å., TANDON, A.K., SIGURDSSON, H. & 5 others (1990). HER-2/*neu* amplification predicts poor survival in node-positive breast cancer. *Cancer Res.*, **50**, 4332.

BRESLOW, N.E. (1975). Analysis of survival data under the proportional hazards model. *Int. Stat. Rev.*, **43**, 45.

BROOKES, S., SMITH, R., CASEY, G., DICKSON, C. & PETERS, G. (1989). Sequence organisation of the human *int-2* gene and its expression in teratocarcinoma cells. *Oncogene*, **4**, 429.

BURGESS, A.W. (1988). Int-1 and int-2: oncogenic proteins, mitogens and morphogens? *BioEssays*, **8**, 40.

CALLAHAN, R. (1989). Genetic alterations in primary breast cancer. *Breast Cancer Res. Treat.*, **13**, 191.

CASEY, G., SMITH, R., MCGILLIVRAY, D., PETERS, G. & DICKSON, C. (1986). Characterisation and chromosome assignment of the human homolog of *int-2*, a potential proto-oncogene. *Mol. Cell. Biol.*, **6**, 502.

COX, D.R. (1972). Regression models and life-tables. *J. Roy. Stat. Soc.* (B), **34**, 187.

DELLI BOVI, P., CURATOLA, A.M., KERN, F.G., GRECO, A., ITTMANN, M. & BASILICO, C. (1987). An oncogene isolated by transfection of Kapsosi's sarcoma DNA encodes a growth factor that is a member of the FGF family, *Cell*, **50**, 729.

DICKSON, C. & PETERS, G. (1987). Potential oncogene product related to growth factors. *Nature*, **326**, 833.

DIXON, W.J. (1988). *BMDP statistical software*. Berkeley, California: University of California Press.

FANTL, V., BROOKES, S., SMITH, R. & 5 others (1989). Characterisation of the proto-oncogene int-2 and its potential for the diagnosis of human breast cancers. In *Cancer Cells 7*, Furth M & Greaves, M. (eds) p 283. Cold Spring Harbor Press: New York.

FERNÖ, M., BORG, Å. & NORGREN, A. (1983). A comparison of two steroid receptor assays in breast cancer: dextran coated charcoal and isoelectric focusing. *Anticancer Res.*, **3**, 243.

HATADA, I., TOKINO, T., OCHIYA, T. & MATSUBARA, K. (1988). Co-amplification of integrated hepatitis B virus DNA and transforming gene *hst-1* in a hepatocellular carcinoma. *Oncogene*, **3**, 537.

KAPLAN, E.L. & MEIER, P. (1958). Nonparametric estimation from incomplete observations. *J. Am. Stat. Assoc.*, **53**, 457.

KRIEG, P., AMTMANN, E. & SAUER, G. (1983). The simultaneous extraction of high molecular weight DNA and of RNA from solid tumours. *Anal. Biochem.*, **134**, 288.

LAW, M.L., KAO, F.T., WEI, Q. & 8 others (1987). The progesterone receptor gene maps to human chromosome band 11q13, the site of the mammary oncogene *int-2*. *Proc. Natl. Acad. Sci. USA*, **84**, 2877.

LIDEREAU, R., CALLAHAN, R., DICKSON, C., PETERS, G., ESCOT, C. & ALI, I.U. (1988). Amplification of the *int-2* gene in primary human breast tumours. *Oncogene Res.*, **2**, 285.

LISCIA, D.S., MERLO, G.R., GARRETT, C., FRENCH, D., MARIANAI-COSTATINI, R. & CALLAHAN, R. (1989). Expression of *int-2* mRNA in human tumours amplified at the *int-2* locus. *Oncogene*, **4**, 1219.

MANTEL, N. (1966). Evaluation of survival data and two new rank order statistics arising in its consideration. *Cancer Chemother. Rep.*, **50**, 163.

MATTEI, M.-G., KRUST, A., STROPP, U., MATTEI, J.-F. & CHAMBON, P. (1988). Assignment of the human progesterone receptor to the q22 band or chromosome 11. *Hum. Genet.*, **78**, 96.

NAKAMURA, Y., LARSSON, C., JULIER, C. & 11 others (1989). Localisation of the genetic defect in multiple endocrine neoplasia type I within a small region of chromosome 11. *Am. J. Hum. Genet.*, **44**, 751.

NORGREN, A., BORG, Å., FERNÖ, M., JOHANSSON, U., LINDAHL, B. & TSIOBANELIS, K. (1982). Improved method for assay of estradiol and progesterone receptors with special reference to breast cancer. *Anticancer Res.*, **2**, 315.

NUSSE, R. (1988*a*). The activation of cellular oncogenes by proviral insertion in murine mammary cancer. In *Breast Cancer: Cellular and Molecular Biology*, Lippman, M.E. & Dickson, R.B. (eds). p. 283, Kluwer Academic Publishers: Boston.

NUSSE, R. (1988*b*). The *int* genes in mammary tumorigenesis and in normal development. *Trends in Genet.*, **4**, 291.

PATERNO, G.D., GILLESPIE, L.L., DIXON, M.S., SLACK, J.M.W. & HEATH, J.K. (1989). Mesoderm-inducing properties of INT-2 and kFGF: two oncogene-encoded growth factors related to FGF. *Development*, **106**, 79.

PETERS, G., LEE, A.E. & DICKSON, C. (1984). Activation of cellular gene by mouse mammary tumour virus may occur early in mammary tumour development. *Nature*, **309**, 273.

PETERS, G., BROOKES, S., SMITH, R., PLACZEK, M. & DICKSON, C. (1989). The mouse homolog of the *hst/k-FGF* gene is adjacent to *int-2* and is activated by proviral insertion in some virally induced mammary tumors. *Proc. Natl Acad. Sci. USA*, **86**, 5678.

ROUSSEAU-MERCK, M.F., BERNHEIM, A., CHERIF, D. & 5 others (1987). Localisation of the human progesterone receptor gene (PGR) to chromosome 11q22-q23. *Cytogenet. Cell Genet.*, **46**, 685.

SAKAMOTO, H., MORI, M., TAIRA, M. & 6 others (1986). Transforming gene from human stomach cancers and a noncancerous portion of stomach mucosa. *Proc. Natl Acad. Sci. USA*, **83**, 3997.

SCHIMKE, R.T. (1984). Gene amplification, drug resistance, and cancer. *Cancer Res.*, **44**, 1735.

SIGURDSSON, H., BALDETORP, B., BORG, Å. & 4 others (1990). Indicators of prognosis in node-negative breast cancer. *N. Engl. J. Med.*, **322**, 1045.

SOUTHERN, E.M. (1975). Detection of specific sequences among DNA fragments separated by gel electrophoresis. *J. Mol. Biol.*, **98**, 503.

TERADA, M., YOSHIDA, T., MIYAGAWA, K., SAKAMOTO, H. & SUGIMURA, T. (1989). Transforming growth factor gene *hst-1*. In *Cancer Cells 7*, Furth, M. & Greaves, M. (eds). p. 311. Cold Spring Harbor Press: New York.

THEILLET, C., LE ROY, X., DE LAPEYRIÈRE, O. & 8 others (1989). Amplification of *FGF*-related genes in human tumours: possible involvement of *HST* in breast carcinomas. *Oncogene*, **4**, 915.

THOMAS, K.A. (1988). Transforming potential of fibroblast growth factor genes. *Trends in Biochem. Sci.*, **13**, 327.

TSUDA, T., NAKATANI, H., MATSUMURA, T. & 7 others (1988). Amplification of the hst-1 gene in human Oesophageal carcinomas. *Jpn. J. Cancer Res.*, **79**, 584.

TSUDA, H., HIROHASHI, S., SHIMOSATO, Y. & 11 others (1989). Correlation between long-term survival in breast cancer patients and amplification of two putative oncogene-coamplification units: *hst*-1/*int*-2 and *c-erb*B-2/*ear*-1. *Cancer Res.*, **49**, 3104.

TSUJIMOTO, Y., YUNIS, J., ONORATO-SHOWE, L., ERIKSON, J., NOWELL, P.C. & CROCE, C.M. (1984). Molecular cloning of the chromosomal breakpoint of B-cell lymphomas and leukemias with the t(11;14) chromosome translocation. *Science*, **224**, 1403.

TSUTSUMI, M., SAKAMOTO, H., YOSHIDA, T. & 4 others (1988). Coamplification of the *hst*-1 and *int*-2 genes in human cancers. *Jpn. J. Cancer Res.*, **79**, 428.

VARLEY, J.M., WALKER, R.A., CASEY, G. & BRAMMAR, W.J. (1988). A common alteration to the *int*-2 proto-oncogene in DNA from primary breast carcinomas. *Oncogene*, **3**, 87.

WADA, A., SAKAMOTO, H., KATOH, O. & 5 others (1988). The homologous oncogenes, *HST1* and *INT2*, are closely located in human genome. *Biochem. Biophys. Res. Commun.*, **157**, 828.

WAHL, G.M. (1989). The importance of circular DNA in mammalian gene amplification. *Cancer Res.*, **49**, 1333.

WILKINSON, D.G., PETERS, G., DICKSON, C. & MCMAHON, A.P. (1988). Expression of the FGF-related proto-oncogene *int-2* during gastrulation and neurulation in the mouse. *EMBO J.*, **7**, 691.

YOSHIDA, T., MIYAGAWA, K., ODAGIRI, H. & 4 others (1987). Genomic sequence of *hst*, a transforming gene encoding a protein homologous to fibroblast growth factors and the *int*-2-encoded protein. *Proc. Natl. Acad. Sci. USA*, **84**, 7305.

YOSHIDA, M.C., WADA, M., SATOH, H. & 8 others (1988*a*). Human *HST1* (*HSTF1*) gene maps to chromosome band 11q13 and coamplifies with the *INT2* gene in human cancer. *Proc. Natl Acad. Sci. USA*, **85**, 4861.

YOSHIDA, T., MURAMATSU, H., MURAMATSU, T. & 4 others (1988*b*). Differential expression of two homologous and clustered oncogenes, *hst*1 and *int*-2, during differentiation of F9 cells. *Biochem. Biophys. Res. Commun.*, **157**, 618.

ZHOU, D.J., CASEY, G. & CLINE, M.J. (1988). Amplification of human *int*-2 in breast cancers and squamous carcinomas. *Oncogene*, **2**, 279.